# Assessment of emergency care services in Nigerian hospitals: A cross-sectional study

**DOI:** 10.1016/j.afjem.2025.100939

**Published:** 2026-01-29

**Authors:** Kelechi Umoga, Muzzammil Muhammad, Melissa A. Meeker, Jessica Rayo, Kehinde Olawale Ogunyemi, Christine Ngaruiya

**Affiliations:** a Harvard Medical School, Massachusetts General Hospital/Brigham Women’s Hospital, 2 Bowdoin Street Everett, MA, 02149, USA; b Beth Israel Deaconess Medical Center, 330 Brookline Ave, Boston, MA, 02215, USA; c Massachusetts General Hospital, 125 Nashua Street, Boston, MA, USA; d Stanford University School of Medicine, Palo Alto CA, 900 Welch Road Suite 350 CA 94304, USA; e Babcock University Teaching Hospital, Department of Community Medicine, Ilisham-Remo, Ogun State, Nigeria

**Keywords:** Emergency care, Nigeria, In-hospital, Capacity, Assessment

## Abstract

**Background:**

The Accident and Emergency (A&E) unit is a key entry point for healthcare in Nigeria, yet data on its functionality and capacity for emergency care remain limited. This study evaluates the functional capacity of A&E units in Nigeria using a modified Emergency Care Assessment Tool (ECAT).

**Methods:**

A cross-sectional study was conducted from September to October 2020 in seven tertiary hospitals across Nigeria. Six hospitals, one from each of the country’s six geopolitical zones, were randomly selected, while the seventh-Nigeria’s main trauma center located in the capital-was purposively included. Doctors and nurses routinely working in A&E units were interviewed using a modified Emergency Care Assessment Tool (ECAT), which evaluates the availability of essential medical interventions (signal functions) for managing six common, life-threatening sentinel conditions: Maternal and Child emergencies, Respiratory failure, Trauma, Shock, Altered mental status, and Severe pain.

**Results:**

Among 503 healthcare providers surveyed (393 doctors and 110 nurses), significant differences were observed in the performance of signal functions across all six sentinel conditions (*p* < 0·001) and across the seven study sites (*p* < 0·001). The overall average capacity score was 2·69 out of 3. Federal Medical Center Katsina (North-West zone) recorded the highest mean score of 2·92 (95% CI: 2·77 - 3·07), while UCTH (South-South zone) had the lowest at 2.44 (95% CI: 2·27 - 2·60). Among the conditions assessed, respiratory failure had the lowest mean score at 2·55 (1·88–3·21).

**Conclusion:**

This study reveals a higher-than-expected national average A&E capacity score (2·69 out of 3) in Nigerian tertiary hospitals but highlights ongoing challenges, particularly in managing respiratory emergencies. Notable regional disparities were observed, with the Northern region outperforming others. Findings emphasize the need for objective, on-site evaluations and broader inclusion of facilities, along with targeted, region-specific investments to improve equitable emergency care nationwide.

## African Relevance

• This study is the first national functional capacity assessment of Adult Emergency Departments in Nigeria, moving beyond prior studies focused only on mortality.
• Conducted in Africa’s most populous country, the findings offer valuable insights to guide targeted improvements in emergency care.
• The results highlight growing recognition of emergency care infrastructure and signal progress toward stronger, more equitable emergency care systems across Nigeria and the broader African continent.

## Introduction

Emergency care systems affect mortality and morbidity at all levels of society [1]. The World Health Organization (WHO) highlights emergency care systems as the “first point of contact with the health system” and emphasizes the importance of “development of quality, timely emergency care accessible to all” [2]. However, there remain significant barriers in access to emergency care in Low- and Middle-income Countries (LMICs) [3,4] especially South-East Asia and Africa with the highest burden of deaths and disability due to infectious and non-infectious emergent medical conditions [5]. This highlights the importance of developing emergency care to reduce morbidity and mortality in such regions.

Recognizing this need, African countries have made increasing investments in developing emergency care systems, including the establishment of Emergency Medicine (EM) specialist training programs, with EM training programs now established in at least 12 African countries [6]. Much of this progress may be attributed to the efforts of the African Federation for Emergency Medicine (AFEM), which has provided a valuable platform for information exchange among practitioners and experts in Africa [7]

Despite these encouraging trends in Africa, Nigeria’s progress has lagged [8]. The National Postgraduate Medical College of Nigeria published the first curriculum for emergency training in Nigeria in 2020, with the training program at the University College Hospital in Ibadan receiving its initial two-year accreditation only in 2022 [9,10]. This is noteworthy given that Nigeria is Africa’s most populous country (206 million) and shares a significant proportion of the global deaths and disability due to medical emergencies [5,11]. Currently, among the 193 countries of the world, Nigeria has the second highest rate of Road Traffic Injuries (RTIs) [12]. Moreover, the effects of the insurgency have been reported to have reversed years of investment in health care in Nigeria and significantly contributed to Nigeria’s failure to achieve the Millennium Development Goals (MDGs) [13]. During the COVID-19 pandemic, emergency respiratory care became increasingly important due to the high mortality rates associated with acute respiratory distress syndrome [14]. A study assessing Nigeria's preparedness for the pandemic revealed that tertiary hospitals had an average of only four ventilators each [15].

These challenges highlight the need for this study which evaluates the readiness of Accident and Emergency (A&E) units in Nigeria to respond to medical emergencies. There is a lack of comprehensive data on hospital emergency care capacity in Nigeria and existing data is often qualitative and not representative. To fill this knowledge gap, a modified version of the Emergency Care Assessment Tool (ECAT) [16] was used to assess the functional capacity of A&E units in Nigeria. Although the ECAT has been integrated into the WHO Hospital Emergency Unit Assessment Tool (HEAT) for wider geographical applicability beyond Africa [17,18], at the time of this study (2020), the HEAT was undergoing major revisions related to the COVID-19 pandemic and was not yet ready for use. Given fixed timelines for data collection and analysis within the funding period for this study, the ECAT was modified using the emerging HEAT scoring structure to maintain alignment with the evolving WHO emergency care assessment framework. While this adapted version of the tool has not been previously validated or piloted, the study provides highly informative incipient data on the current functional capacity of tertiary A&Es in Nigeria.

## Methods

### Study design and setting

A stratified sampling technique was used in randomly selecting the hospitals for this study. All 42 government-run tertiary hospitals in Nigeria were stratified by the six geo-political zones (administrative areas into which Nigeria is divided). Using a randomization site (www.random.org), one tertiary hospital was selected from the tertiary hospitals in each of the geo-political zones. In addition to six randomly selected facilities per zone, the National Hospital in the Federal Capital Territory was purposively chosen because it hosts Nigeria’s only dedicated National Trauma Center and is the sole Nigerian center accredited for advanced trauma training [19,20].

Fig. 1.

**Fig. 1:**
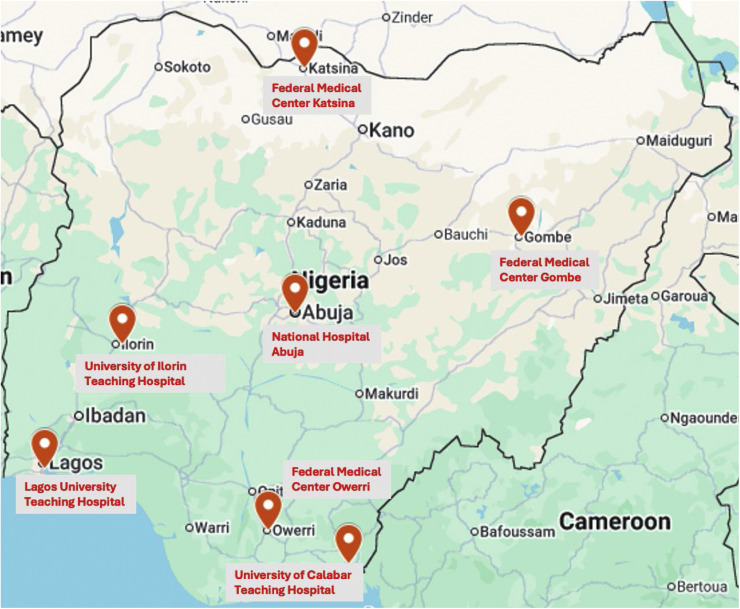
Map of Nigeria with locations of facilities sampled.

### Inclusion criteria and sampling strategy

This study included physicians who had worked in the A&E unit for at least two days per week in the prior three months. For data triangulation, a convenience sample of nurses was included. Nurses were eligible if they were permanent A&E staff and participants were selected based on availability. Data collection occurred between September 1 and October 5, 2020, during interview hours (10:00 AM to 5:00 PM, Monday to Saturday).

### Instruments and measures

This study utilized a modified version of the Emergency Care Assessment Tool (ECAT), designed to evaluate emergency department functionality focusing on 73 key interventions (signal functions) for six critical (sentinel) conditions: Trauma, Respiratory failure (RESP), Altered mental status (AMS), Shock, Severe pain (PAIN), and maternal and child emergencies (MCH) [16]. The ECAT was modified in two key ways. First, we incorporated a three-point scoring system: “Generally Not Done,” “Sometimes Done,” and “Always Done,” adapted from the WHO Hospital Emergency Unit Assessment Tool (HEAT) [18]. This expanded on the ECAT’s original binary scoring, which classified signal functions as “Yes” (≥90 % of the time) or “No” (<90 %) [21]. Second, we included additional context-specific questions on provider demographics, employment status, and payment.

### Data collection

An electronic version of the adapted ECAT was uploaded to KoboToolbox (version 2.020.30). Following informed consent, research assistants conducted in-person interviews, at the end of healthcare providers’ shifts.

### Statistical analyses

Survey data were analyzed using the R statistical software (version 4.0.4) package. Responses were analyzed to determine distribution patterns across hospital sites and by sentinel condition. For each signal function, responses of “Generally Not Done,” “Sometimes Done,” and “Always Done” were coded as 1, 2, and 3, respectively. Within each survey, scores for signal functions corresponding to a single sentinel condition were averaged to yield a condition-specific performance score. The six condition scores were then averaged to produce an overall emergency care performance score for each survey (or provider). Mean scores and 95 % CIs were calculated nationally and by site for sentinel conditions and overall performance. To assess differences in performance, a two-way ANOVA was used to compare scores across the six sentinel conditions and the seven hospital sites. Independent *t*-tests were conducted to compare each site's overall performance score to the ideal score of 3.0 (“Always Done”) and to the national mean, evaluating both absolute deviation from the ideal and regional disparities. To address potential bias due to varying respondent numbers across sites, we conducted a sensitivity analysis by randomly sampling 50 surveys per site, and recalculated performance scores and repeated the *t*-tests to assess the robustness of our findings.

### Ethical consideration

Ethical approval was obtained from the Yale Human Research Protection Program Institutional Review Board (2000,027,053), the National Health Research Ethics Committee of Nigeria (NHREC/01/01/2007–29/09/2019), and the Ethical Review boards of each of the seven tertiary hospitals involved in the study.

## Results

A total of 503 health care providers were interviewed across all seven facilities (Table 1). Of the 503 healthcare providers surveyed, 390 were doctors, 110 were nurses, and 3 did not indicate their role. The doctors interviewed were at different levels in their careers and training. Doctors undergoing their residency training program (registrars and senior registrars) constituted the majority of the respondents, while the doctors undergoing their internship (interns or house officers) were in the minority.

**Table 1:** Survey respondents by site and provider type.

Facility [Geo-political Zone]	Providers (n) %	Provider Type						
		Nurse	Registrar^a^ (Residents)	Medical Officer^b^ (working full-time in the A&E)	Medical Officer (working part-time in the A&E)	Senior Registrar^c^ (Senior resident)	National Youth Service Corps Members^d^	Other^e^
Federal Medical Center, Katsina [North-West]	102 (20.3)	49	20	24	6	1	0	2
Lagos University Teaching Hospital [South-West]	75 (14.9)	0	63	14	1	0	0	0
University of Calabar teaching Hospital [South-South]	76 (15.1)	13	31	6	7	0	14	5
Federal Teaching Hospital Gombe [North-East]	70 (13.9)	9	18	6	19	17	0	1
University of Ilorin Teaching Hospital [North-Central	67 (13.3)	11	11	21	12	6	0	3
Federal Medical Center Owerri [South-East]	63 (12.5)	12	32	4	1	11	0	3
National Hospital Abuja [North Central]	50 (9.9)	16	21	1	4	4	2	2
Total	503	110	196	76	50	39	16	16

a Junior residents in any given specialty.

b Doctors who have not undergone residency training (but have completed both internships and mandatory national service program (see below).

c Senior residents in any given specialty.

d Doctors who have completed their mandatory internship and are now completing a mandatory year of service required for all Nigerian graduates upon graduation irrespective of field.

e Other consultants and those that did not provide a specific clinical role .

Of the three possible scores pertaining to each signal function within a sentinel condition, a score of '3’ had the highest frequency of responses across all the conditions (Table 2). Of all the six sentinel conditions assessed, respiratory failure had the lowest mean score of 2·55 (95 % C.I. 1·88 - 3·21), indicating that the sites were least equipped to manage respiratory failure (see Table 3). Conversely, altered mental status (AMS) had the highest score of 2·77 (95 % C.I, 2·36 - 3·19), followed by shock and severe pain with the same mean score of 2·74 and 95 % C.I of (2·26 - 3.22) and (2·13 - 3.34) respectively. Maternal and child emergencies (MCH) and trauma tied with the 3rd highest mean score of 2·67 (95 % C.I, 1·60 - 3·73) and (95 % C.I, 2·22 - 3·13), respectively (Table 2).

**Table 2:** Average frequency of responses and mean scores across all sites stratified by sentinel condition.

	Score					
	1	2	3	N/A^a^^,^^b^		
Sentinel Condition	N				Total	Mean Score [95 % CI]
MCH^a^	44	73	369	17	503	2·67 [1·60 - 3·73]
TRAUMA	46	69	377	11	503	2·67 [2·22 - 3·13]
RESP^a^	72	80	346	4	503	2·55 [1·88 - 3·21]
AMS^a^	47	19	435	3	503	2·77 [2·36 - 3·19]
SHOCK	35	61	406	2	503	2·74 [2·26 - 3·22]
PAIN	45	41	413	5	503	2·74 [2·13 - 3·34]

a N/A Not Applicable; MCH, Maternal and Child Emergencies; RESP, Respiratory failure; AMS, Altered Mental Status; PAIN, Severe Pain.

b N/A: Average number of respondents, who responded to less than half of the signal function questions under a given sentinel condition.

**Table 3:** Average frequency of responses and mean score across all conditions stratified by site.

	Score					
	1	2	3	N/A^a^^,^^b^		
Sites (Geo-political zone)	N				Total	Mean Score
aNHA (North central)	6	3	36	4	49	2·67 [2·30 - 3·05]
aUCTH (South-South)	12	18	46	0	76	2·44 [2·27 - 2·60]
aFMC Gombe (North-East)	5	18	47	0	70	2·60 [2.38 - 2·81]
aFMC Owerri (South-East)	10	2	51	1	64	2·66 [2·13 - 3·19]
aFMC Katsina (North-West)	2	4	96	0	102	2·92 [2·77 - 3·07]
aUITH (North-Central)	10	4	53	0	67	2·64 [2·49 - 2·80]
aLUTH (South-West)	3	9	62	1	75	2·80 [2·55 - 3·05]
All sites	48	57	391	7	503	2.69 [2·28 - 3·10]

a N/A Not Applicable; NHA, National Hospital Abuja; UCTH, University of Calabar Teaching Hospital; FMC, Federal Medical Center; UITH,University of Ilorin Teaching Hospital; LUTH, Lagos University Teaching Hospital.

b N/A: Average number of respondents, who responded to less than half of the signal function questions for each given site.

Of the three possible scores pertaining to each signal function assessed per site, a score of '3’ had the highest frequency of responses across all the sites (Table 3). Of all the seven sites studied, FMC Katsina which represents the north-west geopolitical zone had the highest mean score of 2·92 (95 % C.I [2·77, 3·07]), while UCTH representing the south-south had the lowest mean score of 2·44 (95 % CI [2·27, 2·60]) (see Table 4). National Hospital Abuja (the purposively selected site with a dedicated Trauma center) had a mean score of 2·67 (95 % CI [2·30, 3·05]) (Table 3).

**Table 4:** Mean Scores by sentinel condition and site.

	Site							
	National (n = 503)	aLUTH, Lagos State (n = 75)	aFMC Owerri, Imo State (n = 63)	aFMC Katsina, Katsina State (n = 102)	aUITH, Kwara State (n = 67)	aFMC Gombe, Gombe State (n = 70)	aUCTH, Cross River State (n = 76)	aNHA, Abuja (n = 50)
Sentinel Condition	Mean Score							
All Conditions	2·69	2·80 (p < 0·001)	2·66 (p = 0·377)	2·92 (p < 0·001)	2·64 (p < 0·001)	2·60 (p < 0·001)	2·44 (p < 0·001)	2·67 (p = 0·563)
aRESP	2·55	2·82	2·64	2·86	2·33	2·44	2·07	2·56
aMCH	2·67	2·35	2·29	2·99	3·00	2·33	2·89	2·64
TRAUMA	2·67	2·72	2·78	2·84	2·71	2·44	2·49	2.71
SHOCK	2·74	2.97	2·81	2·89	2·63	2·81	2·40	2·57
aPAIN	2·74	2.98	2·72	2·98	2·58	2·82	2.24	2.76
aAMS	2·77	2.95	2·71	2·96	2·63	2·75	2·55	2·78

a MCH, Maternal and Child Emergencies; RESP, Respiratory failure; AMS, Altered Mental Status; PAIN, Severe Pain; NHA, National Hospital Abuja; UCTH, University of Calabar Teaching Hospital; FMC, Federal Medical Center; UITH,University of Ilorin Teaching Hospital; LUTH, Lagos University Teaching Hospital.

A two-way ANOVA test indicated that both site (*p* < 0.001) and sentinel condition (*p* < 0.001) are related to significant differences in scores. The mean score of 2·69 provides a snapshot of overall capacity from this national sampling of sites, out of the reference score of 3 (Table 4). Independent *t*-tests comparing the site-specific overall performance scores to the maximum score of ‘3′ showed that all sites differed significantly from this maximum score (*p* < 0.001 for each site). Additionally, the distribution of each individual sentinel condition performance score across all surveys significantly differed from the maximum score of ‘3’ (*p* < 0.001 for each condition). Furthermore, independent T-tests indicated that the site-specific overall performance scores for LUTH, FMC Katsina, UITH, FMC Gombe, and UCTH were significantly different compared to the national distribution of overall performance scores (*p* < 0.001 for each listed site) (Table 4). However, the site-specific overall performance scores for FMC Owerri and NHA did not significantly differ from the national distribution of overall performance scores (*p* = 0.377 and *p* = 0.563, respectively) (Table 4).

A sensitivity analysis was performed using 50 randomly selected responses from each site to determine if the national average scores were biased by the variation in the number of study respondents across sites. The national average overall performance score was insignificantly different (2.69 in the primary analysis compared to 2.68 in the sensitivity analysis) (Table 4 and Appendix). Furthermore, the results from the independent T-tests comparing the site-specific distributions of overall performance scores to the national distribution largely redemonstrated statistically significant differences seen in the primary analysis. There was one exception of the site (UITH), where a statistical difference from the National Average score previously noted was not re-demonstrated in the sensitivity analysis.

## Discussion

This study represents the first national assessment of adult emergency care capacity in tertiary hospitals across Nigeria, Africa’s most populous country [11].We found a national average emergency care capacity score of 2·69 out of 3. This score, derived from the modified ECAT, suggests that critical emergency functions are available often but are still not performed consistently across all cases and conditions. Among sentinel conditions assessed, capacity to manage respiratory failure was the lowest (2·55), while altered mental status scored highest (2·77). These differences, along with regional score variations, were statistically significant (*p* < 0.001), with FMC Katsina achieving the highest individual facility score of 2·92.

Although this average falls short of the ideal (a consistent score of 3), it is higher than previously reported figures in the limited literature on Nigerian emergency care. Prior studies have painted a bleaker picture. A 2021 qualitative study highlighted a pervasive sense of vulnerability among emergency care providers [22], while a cross-sectional analysis of pediatric emergency departments reported medication and equipment performance scores of 50.7 % and 43.9 %, respectively [23]. Additionally, some data suggest that 24-hour A&E mortality in Nigeria is higher than the average in low- and middle-income countries (LMICs) [[24], [25], [26]]. The relatively higher capacity scores in our study could be influenced by response bias, with respondents possibly overstating their facility’s capabilities. As data were self-reported through interviews, social desirability may have played a role. Future research should include site visits and direct observation to validate reported capacities. Despite potential reporting bias, the improved scores may also reflect long-term investment in emergency care infrastructure and training, supported by partnerships with high-income countries and collaborations within Africa [27]. These initiatives may have contributed to stronger systems than previously documented, though gaps clearly remain.

The significant differences in performance across sentinel conditions suggest variability in how well facilities manage different emergencies. This pattern is consistent with findings from Nepal and Eswatini, where some emergency conditions were more reliably managed than others, largely due to training deficits and equipment shortages [18,28]. In our study, the low capacity for respiratory failure management is especially concerning, given the burden of COVID-19, which caused significant mortality due to respiratory complications [29]. Evidence from India - a similarly densely populated country - shows that early intubation improved COVID-19 outcomes [30] yet Nigeria’s emergency preparedness remains limited, with only about four ventilators per tertiary hospital on average [15]. Human resource limitations further compound this issue, with only one registered pulmonary or critical care physician per 2.3 million people [31]. Given that data collection took place during the COVID-19 pandemic - a period marked by staffing shortages and equipment disruptions - participants' responses regarding respiratory care could have been influenced by these circumstances. These gaps underscore the urgent need for targeted investment in both equipment and workforce to improve emergent respiratory conditions-critical not only for pandemics but also for seasonal and endemic respiratory illnesses.

Regional disparities were another key finding. While FMC Katsina in the northwest scored highest, UCTH in the south-south had the lowest score (2.44). This finding is in contrast to previous studies indicating poorer healthcare with regards to equipment availability in Northern Nigeria [23], attributed in part to the impact of regional conflicts [32]. For instance, research has consistently shown the North to lag behind in maternal care utilization and health outcomes [33]. However, several factors may explain the unexpectedly high performance at FMC Katsina. First, efficient use of limited resources may account for the higher score despite constrained funding. Second, our study focused on adult emergency departments, which may be better resourced than pediatric units assessed in earlier research [23]. Lastly, targeted investments-such as Katsina’s Mobile Ambulance Service launched in 2008 to bring care to underserved rural areas-may have contributed to stronger health infrastructure at the facility [34]. Given the possibility that this could very well be a site-specific factor instead of a generalizable regional trend, the findings should be interpreted with caution. Therefore, nationally representative assessments are recommended to better understand and track regional disparities and improvements over time, particularly in areas affected by conflict or chronic underfunding.

The findings from the National Hospital Abuja (NHA), Nigeria’s only tertiary hospital with a dedicated trauma center, also warrant discussion. Contrary to expectations, NHA did not rank highest in trauma care, placing fifth among surveyed sites. This result challenges assumptions that infrastructure alone equates to superior performance. Possible explanations include overestimation of capacity by other sites due to social desirability bias or the NHA being overwhelmed by high patient volumes from its large catchment area. This suggests that while infrastructure is essential, it must be supported by adequate staffing, resource availability, and efficient patient flow systems to ensure effective emergency care delivery.

### Limitations

The convenience sampling of nurses may have overrepresented certain nurse perspectives and excluded night-time staff possibly skewing the responses. Additionally, social desirability bias may have influenced responses, but efforts such as using neutral data collectors, and conducting private interviews helped minimize this risk. Including direct observation of the A&E practices will be useful in mitigating this bias in the future. Furthermore, Generalizability is constrained by the relatively small number of sites, focus on tertiary hospitals and exclusion of rural/secondary facilities where most Nigerians receive care. Thus, we would caution against extrapolating findings to the broader health system. Also, to our knowledge, this modified version of the ECAT used has not been previously validated or piloted which limits comparability with prior ECAT studies. Lastly, this study was obtained in 2020 (5 years ago) at the peak of pandemic, thus given the changes to the field and to the country since then, more recent insights will be a useful complement to this study.

## Conclusion

This study provides meaningful insights into the functional capacity of adult accident and emergency (A&E) units in Tertiary hospitals in Nigeria especially in light of sparse literature in this space currently. While the national average A&E capacity score of 2.69 out of 3 was higher than expected based on previous literature, significant challenges remain especially with capacity for managing respiratory emergencies compared to other conditions. Regional disparities in capacity were also noted with the Northern region performing better than the others. This study highlights the need for more rigorous, objective evaluations including on-site evaluations and inclusion of lower-level hospitals to better understand regional disparities. Our findings also underscore the importance of targeted region-specific investments to ensure equitable emergency care across Nigeria.

## Dissemination of results

The findings of this study were presented at the 6th African Conference of Emergency Medicine (AfCEM 2022) held in Accra Ghana. The findings have also since been shared with colleagues in Nigeria.

## Declaration of generative AI and AI-assisted technologies in the manuscript preparation process

During the preparation of this work the authors used ChatGPT in order to reword sentences in the manuscript, cover letter and response letter to facilitate brevity, improve readability and comprehensibility. After using ChatGPT, the author(s) reviewed and edited the content as needed and take full responsibility for the content of the published article.

## CRediT authorship contribution statement

**Kelechi Umoga:** Conceptualization, Funding acquisition, Methodology, Investigation, Project administration, Resources, Writing – original draft, Writing – review & editing. **Muzzammil Muhammad:** Data curation, Formal analysis, Software, Visualization, Writing – review & editing. **Melissa A. Meeker:** Data curation, Formal analysis, Software, Visualization. **Jessica Rayo:** Writing – review & editing. **Kehinde Olawale Ogunyemi:** Conceptualization, Methodology, Supervision, Validation, Writing – review & editing. **Christine Ngaruiya:** Conceptualization, Methodology, Supervision, Validation, Writing – review & editing.

## Declaration of competing interest

The authors declare that they have no known competing financial interests or personal relationships that could have appeared to influence the work reported in this paper.

## References

[1] Hirshon J.M., Risko N., Calvello E.J., Stewart de Ramirez S., Narayan M., Theodosis C. (2013). Health systems and services: the role of acute care. Bull World Health Organ.

[2] World Health Organization (2018). http://www.who.int/emergencycare/systems/en/.

[3] Reynolds T.A., Bisanzo M., Dworkis D., Hansoti B., Obermeyer Z., Seidenberg P. (2013). Research priorities for data collection and management within global acute and emergency care systems. Acad Emerg Med.

[4] Calvello E., Reynolds T., Hirshon J.M., Buckle C., Moresky R., O’Neill J. (2013). Emergency care in sub-Saharan Africa: results of a consensus conference. Afr J Emerg Med.

[5] Chang C.Y., Abujaber S., Reynolds T.A., Camargo C.A., Obermeyer Z. (2016). Burden of emergency conditions and emergency care usage: new estimates from 40 countries. Emerg Med J.

[6] Sawe H.R., Akomeah A., Mfinanga J.A., Runyon M.S., Noste E. (2019). Emergency medicine residency training in Africa: overview of curriculum. BMC Med Educ.

[7] Stein C., Mould-Millman N.K., De Vries S., Wallis L. (2016). Access to out-of-hospital emergency care in Africa: consensus conference recommendations. Afr J Emerg Med.

[8] DaCosta A., Osonuga A., Adesegun O. (2020). The urgent need for postgraduate medical training in emergency medicine in Nigeria. Afr J Emerg Med.

[9] Udofia O. National postgraduate medical college of Nigeria residency training curriculum and guidelines2020: [93 p]. Available from: http://npmcn.edu.ng/downloads/APPROVED%20EMERGENCY%20MEDICINE%20CURRICULUM-%202020.pdf.

[10] Olaomi O.O., Okoye G.O., Balarabe-Musa B. (2024). Current trends in emergency medicine in Nigeria. Niger J Clin Pr.

[11] Adesola R.O., Opuni E., Idris I., Okesanya O.J., Igwe O., Abdulazeez M.D. (2024). Navigating Nigeria's health landscape: population growth and its health implications. Env Health Insights.

[12] Labinjo M., Juillard C., Kobusingye O.C., Hyder A.A. (2009). The burden of road traffic injuries in Nigeria: results of a population-based survey. Inj Prev.

[13] Oleribe O.O., SD Taylor-Robinson (2016). Before sustainable development goals (SDG): why Nigeria failed to achieve the millennium development goals (MDGs). Pan Afr med j.

[14] Wilcox S.R., Condella A. (2021). Emergency department management of severe hypoxemic respiratory failure in adults with COVID-19. J Emerg Med.

[15] Ogoina D., Mahmood D., Oyeyemi A.S., Okoye O.C., Kwaghe V., Habib Z. (2021). A national survey of hospital readiness during the COVID-19 pandemic in Nigeria. PLoS One.

[16] Bae C., Pigoga J.L., Cox M., Hollong B., Kalanzi J., Abbas G. (2018). Evaluating emergency care capacity in Africa: an iterative, multicountry refinement of the emergency care assessment tool. BMJ Glob Health.

[17] Bredow Z., Corbett Z., Tarawally M.M., Jackson L., Mansaray F.T., Sesay S. (2024). Emergency care capacity in Sierra Leone: a multicentre analysis. Afr J Emerg Med.

[18] Pigoga J.L., Joiner A., Chowa P., Luong J., Mhlanga M., Reynolds T.A. (2020). Evaluating capacity at three government referral hospital emergency units in the kingdom of Eswatini using the WHO hospital emergency unit assessment tool. BMC Emerg Med.

[19] Olaomi O.O. Quality of care policy and operational guidelines: national Hospital Abuja; 2018 [Available from: https://nationalhospital.gov.ng/getting-around-nha/.

[20] Gwaram U.A., Okoye O.G., Olaomi O.O. (2021). Observed benefits of a major trauma centre in a tertiary hospital in Nigeria. Afr J Emerg Med.

[21] Chavula C., Pigoga J.L., Kafwamfwa M., Wallis L.A. (2019). Cross-sectional evaluation of emergency care capacity at public hospitals in Zambia. Emerg Med J.

[22] Usoro A., Aiwonodagbon B., Strong J., Kivlehan S., Akodu B.A., Olufadeji A. (2021). Perspectives on the current state of Nigeria's emergency care system among participants of an emergency medicine symposium: a qualitative appraisal. BMJ Open.

[23] Enyuma C.O.A., Moolla M., Motara F., Olorunfemi G., Geduld H., Laher A.E. (2020). Paediatric emergency department preparedness in Nigeria: a prospective cross-sectional study. Afr J Emerg Med.

[24] Ugare G.U., Ndifon W., Bassey I.A., Oyo-Ita A.E., Egba R.N., Asuquo M. (2012). Epidemiology of death in the emergency department of a tertiary health centre south-south of Nigeria. Afr Health Sci.

[25] Rukewe A., Fatiregun A., Okolo C.A., Ojifinni K., Akinola O., Nweke M.C. (2015). Emergency department deaths in a nigerian university hospital: deaths too many. West Indian Med J.

[26] Obermeyer Z., Abujaber S., Makar M., Stoll S., Kayden S.R., Wallis L.A. (2015). Emergency care in 59 low- and middle-income countries: a systematic review. Bull World Health Organ.

[27] Lecky F.E., Reynolds T., Otesile O., Hollis S., Turner J., Fuller G. (2020). Harnessing inter-disciplinary collaboration to improve emergency care in low- and middle-income countries (LMICs): results of research prioritisation setting exercise. BMC Emerg Med.

[28] Kharel R., Thapa G.B., Voor T., Pant S.R., Adhikari S.K., Bist B.S. (2023). Emergency unit assessment of seven tertiary hospitals in Nepal using the WHO tool: a cross-sectional study. Int J Emerg Med.

[29] Zhang B., Zhou X., Qiu Y., Song Y., Feng F., Feng J. (2020). Clinical characteristics of 82 cases of death from COVID-19. PLoS One.

[30] Zirpe K.G., Tiwari A.M., Gurav S.K., Deshmukh A.M., Suryawanshi P.B., Wankhede P.P. (2021). Timing of Invasive Mechanical Ventilation and Mortality among Patients with severe COVID-19-associated acute respiratory distress syndrome. Indian J Crit Care Med.

[31] Obaseki D., Adeniyi B., Kolawole T., Onyedum C., Erhabor G. (2015). Gaps in capacity for respiratory care in developing countries. Nigeria as a case study. Ann Am Thorac Soc.

[32] Chukwuma A., Ekhator-Mobayode U.E. (2019). Armed conflict and maternal health care utilization: evidence from the Boko Haram Insurgency in Nigeria. Soc Sci Med.

[33] Okoli C., Hajizadeh M., Rahman M.M., Khanam R. (2020).

[34] Peters G., Doctor H., Afenyadu G., Findley S., Ager A. (2014). Mobile clinic services to serve rural populations in Katsina State, Nigeria: perceptions of services and patterns of utilization. Health Policy Plan.

